# Psychosomatic consultation in the workplace: opportunities and limitations of the services offered—results of a qualitative study

**DOI:** 10.1007/s00420-015-1098-y

**Published:** 2015-11-12

**Authors:** Christine Preiser, Eva Rothermund, Andrea Wittich, Harald Gündel, Monika A. Rieger

**Affiliations:** Institute of Occupational and Social Medicine and Health Services Research, University Hospital Tübingen, Tübingen, Germany; Department of Psychosomatic Medicine and Psychotherapy, University Hospital Ulm, Ulm, Germany; Department of Psychosomatic Medicine and Psychotherapy, University Hospital Freiburg, Freiburg im Breisgau, Germany

**Keywords:** Occupational health physician, Workplace, Psychosomatic consultation in the workplace, Common mental disorders, Health services research, Development of a new health-related service

## Abstract

**Purpose:**

In Germany, innovative concepts of anchoring psychotherapeutic consultations within an occupational setting emerge in models like the “psychosomatic consultation in the workplace” (PCIW). Characteristic quality is the close cooperation between company-based occupational health physicians (OPs) and external psychotherapeutic consultants. Little is currently known about the attitudes of OPs and other stakeholders in companies in terms of possible contributions of these offers to their tasks within the field of mental health and work.

**Methods:**

Data were collected via individual interviews with different stakeholders (*n* = 8) and two OP focus groups (each *n* = 5) with and without experience with PCIW. Data were analysed with content analysis.

**Results:**

Common mental disorders (CMD) were perceived as occurring increasingly but still being stigmatized. PCIW allows employees quick access to a neutral psychotherapist and thus might help to avoid chronification of CMD. For companies, this may mean that longer periods of absenteeism (and presenteeism) can be avoided. The interviewees also feel that the ongoing collaboration with a psychotherapeutic specialist may sensitize OPs to recognize mental disorders earlier and provide basic treatment. PCIW was stated as an early, easy and fast first access to psychotherapy. The effort of PCIW is limited if structural changes in the workplace are necessary to reduce mental stressors. Also, if financed by the company, PCIW should have clear time limits and cannot aim to replace health insurance benefits.

**Conclusions:**

Taking above-mentioned limitations into account, PCIW appears to be a promising tool to bridge the gap between OP-conducted company-based health promotion and early secondary care.

## Introduction

Mental illnesses are being cited more and more frequently as a reason for sick leave: in 2014, 17 % of sick leave days in Germany were due to common mental disorders (CMD), i.e. mainly depression and anxiety according to the DAK Health Report (Hildebrandt et al. 2014). At the same time, CMD are now the leading cause of premature retirement due to reduced earning capacity—namely in four out of ten employees who drop out of the labour force early (BPtK 2014).

Evidence from OECD reports that draws on national data, OECD data and other international scientific data suggests that about 15 % of the working-age population experiences CMD or mild psychological impairment. Mental disorders are known to reduce actual employment prospects, productivity and wages, but are also a strong predictor for future impaired work functioning and negative clinical outcome (OECD 2012). In general, expenditures on mental health have been rising in industrial countries (OECD 2014). Together with indirect costs (increased unemployment, reduced productivity), CMD stands for significant social and economic costs of more than 4 % of the gross domestic product. Beyond that, international estimates suggest the treatment gap, i.e. the percentage of individuals who require care but do not get it, is around 60 % regarding depression and anxiety disorders, to name two examples (OECD 2014).

In Germany, the statutory health insurance system, which covers more than 90 % of the population, carries the costs of outpatient psychotherapy. Thus, “medical” or “psychological” psychotherapists, meaning medical doctors with a psychiatric or psychosomatic specialization as well as psychologists with training as a “psychological psychotherapist”, mainly provide psychotherapy. Even though health services in Germany are known to provide good outpatient treatment, Germany contributes to the above-described treatment gap: access to outpatient psychosomatic and psychotherapeutic services through statutory standard care is characterized by long waiting periods for initial consultations (up to 2.2 months) and again for the initiation of psychotherapy (up to 4.8 months) (Schulz et al. 2008; Kruse and Herzog 2012).

In addition to general health care, health-related services for employees are offered by occupational health physicians (OPs) in Germany. They work in companies, dealing with both work-related and general health problems of the employees. Their work is paid by the employers. According to occupational health regulations and social security laws, OPs deal with the primary, secondary and tertiary prevention of work-related and general diseases. The work of OPs has several interfaces, e.g. with general practitioners (Moßhammer et al. 2011, 2012, 2014, van Amstel et al. 2005; Rijkenberg et al. 2013; Verger et al. 2014) or rehabilitation (Völter-Mahlknecht and Rieger 2014). They serve as mediators between the different kinds of preventive offers and between inside and outside the company and are gatekeeper between primary and secondary care offers. Yet, their work is not paid by the statutory health insurance but by the employers.

More and more organizations express concern over the extent of mental disorders. Occupational health physicians together with worksite stakeholders address the importance of addressing mental health issues and voice the need for structures to deal with these issues. In particular, large companies have already begun offering specific psychosocial services to their employees in recent years, such as general social and psychosocial counselling (Klein and Appelt 2010), including employee addiction services (Klein and Appelt 2011) and psychological supervision and coaching within leadership (Hibbeler 2012; Moos and Wittich 2012). Some companies have now expanded this range of services to include “psychosomatic consultation in the workplace” (PCIW) (Mayer et al. 2010; Rothermund et al. 2012, 2014; Preiser et al. 2014). This term is used as a collective term for services offered in different companies, each of which is named and structured differently. They all have in common that employees are offered one or several consultations with a psychotherapeutic/psychosomatic specialist—at least for the first 1–5 sessions at the company’s expense—in the case of beginning psychosomatic complaints or an impending mental disorder. The goal is timely detection and early intervention to significantly improve the individual prognosis and, at the same time, to keep the (psychosocial and financial) costs associated with mental health complaints as low as possible for both employees and employers (Wege and Angerer 2013; Burman-Roy et al. 2013). Such approaches go along with experts’ recommendations for early intervention, “of keeping an individual, and their treatment, as close to the workplace as possible” (Henderson et al. 2011).

To date, there is little scientific work investigating the components and interactions of the complex intervention PCIW. Thus, an explorative approach was chosen (Campbell et al. 2000). After describing characteristic components and different healthcare models of PCIW elsewhere (Preiser et al. 2014), the attitudes regarding the intervention of persons concerned are being explored in the present article. Addressing more conceptual questions, we will discuss in this paper the opportunities and limitations that the interview partners see for PCIW against the background of their perspectives on CMD at the workplace.

## Methods

### Study design

Eight individual interviews and two focus group discussions were conducted with experts following an exploratory model. Individual interviews and focus groups are both established methods of data collection in occupational groups. In a group setting, participants mutually prompt each other to engage in in-depth discussions of various topics (Morgan 1988). The definition of “expert” used in this study focuses on the combination of specific knowledge and involvement in problem-solving processes (Meuser and Nagel 2009). In addition to OPs (in focus groups), stakeholders related to companies, i.e. representatives of internal consulting services, personnel departments, company health insurance funds and staff representatives, were approached as experts in individual interviews.

### Study population

Two focus groups were formed as follows: one consisted of OPs with PCIW experience, the other of OPs without such experience. In the individual interviews, structural variation (Patton 1990) was achieved by differentiating between respondents “with PCIW experience” and “without PCIW experience” and between the categories “large enterprise” and “small-/medium-sized enterprise” as well as by mapping various occupational groups. Since all companies are potentially affected by CMD and associated challenges, we decided to include also experts without experience to survey their expectations and concerns regarding a programme like the PCIW. The study sample is shown in Table 1.

**Table 1:** Composition of study population (interview code in parentheses)

*(a) Focus groups (FG): number of interviewees and their fields of work*		
	**Occupational health physicians with experience of PCIW services (FG_AMM-01)**	**Occupational health physicians without experience of PCIW services (FG_AMO-01)**
	5 OPs from various large companies in the metal and electrical or automotive industry (approx. 2500 to >15,000 employees at each site)	3 Freelance OPs who work for several companies (with ≤approx. 500–1000 employees each)
	Experiences as OPs: 5–26 years	1 Company doctor, inter-company service, works for one medical technology company (approx. 3000 employees)
	All OPs working full time	1 Company doctor, inter-company service, works for several companies (with ≤1000 employees)
	Experience with the service: 0, 5–8 years	Experiences as OPs: 4–26 years
		All OPs working full time

*(b) Expert interviews with company stakeholders: professional activity and field of work* ^*a*^		
	**Experience of PCIW services (EM-01, EM-02, EM-03)**	**No experience of PCIW services (EO-01, EO-02, EO-03, EO-04, EO-05)**
Employee representation	–	1 Full-service hospital (approx. 9000 employees)
		Experience in position: 6 years
Company health insurance fund	–	1 Medical technology company (approx. 3200 employees)
		Experience in position: 3 years
Occupational social counselling/psychosocial counselling	1 Metal and electrical industry (approx. 10,500 employees)	1 Working for a large inter-company occupational health service, i.e. many companies, including small- and medium-sized companies
	Experience in position: 13 years	Experience in position: 13 years
	1 Automotive industry (>15,000 employees)	
	Experience in position: 4 years	
Human resources	–	2 Metal and electrical industry companies (approx. 3000 and approx. 9000 employees)
		Experience in position: 4 and 9 years
Company doctor	1 Metal and electrical industry (approx. 10,500 employees)	–
	Experience in position: 22 years	

^a^We decided to only give little information on the sample in order to protect our interview partners. The number of companies offering PCIW is limited, and it would be easy to identify individuals with more detailed information

The OPs with PCIW experience were easy to identify due to the limited number of companies offering PCIW in south-west Germany at the time of the study: five of the seven eligible OPs participated in the focus group, one participated in an individual interview and one cancelled due to time constraints. The discussants’ working experiences as OPs ranged from 5 to 26 years (all of them working full time), and their experiences with the service ranged from 6 months to 8 years, depending on their company. The OPs without PCIW experience were recruited through (teaching) collaborations of the Tübingen Institute of Occupational and Social Medicine and Health Services Research (5/6 participation rate in the focus group; 1 cancellation due to time constraints). The discussants’ working experiences as OPs ranged from 4 to 26 years (all of them working full time). The additional company contacts for the individual interviews were obtained with the help of the OPs interviewed (8/8 participation rate). Various perspectives were included in order to reach data saturation even with a small study population.

### Implementation

The entire project ran from 1 August 2011 to 29 February 2012. All interviews and focus group discussions were conducted by Christine Preiser, who has no conflict of interest. The focus groups were conducted in autumn 2011 (duration 94 and 102 min, respectively). The individual interviews were conducted by telephone with one exception (duration: 19–34 min, average duration 26 min). All interviews were digitally recorded, and the focus groups were also documented on video (videos were deleted after the speakers had been assigned to the interview transcripts). The interviews of the experts without PCIW experience (individual interviews and focus group) were conducted after the focus group involving the OPs with PCIW experience. Two contrasting examples of PCIW were derived from the latter and used as a stimulus in the focus group with experts without PCIW experience, who discussed the (dis)advantages of features of both models and compared them to their own professional experiences.

### Analysis

The data were transcribed, pseudonymized and then analysed by three independent (neutral) persons applying qualitative content analysis (Mayring 2000; Hsieh and Shannon 2005; Schreier 2012). We derived inductive categories from the data through a first summarizing qualitative content analysis (Mayring 2000), keeping potential deductive categories from our guiding questions in mind. We built a coding frame by structuring these categories, successfully tested the coding frame on our material and then applied it to the remaining interviews (Schreier 2012). Content validation was carried out in a workshop in February 2012, to which all interview partners and other representatives of the same professional groups, representatives of employers’ organizations and unions and the relevant state ministry were invited as part of the research project. A total of 26 people participated in the workshop.

### Ethical vote

The ethics committee of the Faculty of Medicine at the University of Tübingen approved the study protocol. All participants were informed about the study in writing and verbally and gave their consent.

## Results

The various concrete structures of PCIW services in different companies have already been presented elsewhere (Preiser et al. 2014; Rothermund et al. 2014). This article focuses on the perspectives on mental illness at the workplace and—against this background—the opportunities and limitations, criticism and assessments pertaining to the service psychosomatic consultation in the workplace. These sections mirror main categories of our coding frame and are filled with paraphrased topics of the respective subcategories. Selected subcategories will be substantiated with quotes to give insights into our material.

All categories will be presented without content additions by the authors. The relevant quotes can be found in Table 2.

**Table 2:** Attitudes towards common mental disorders and psychosomatic consultations at the workplace (PCIW)—categories and exemplary quotes

Main category	Subcategory	Quote
“Perspective on common mental disorders (CMD) in the workplace”	Quote 1: Causes of psychological disorders	“This is a view that we share, namely that we cannot really answer the question in the company where the mental illness or psychological disorder arose. And that is why the approach of our consultation is not to find causes of mental illness but to contribute to mental health.” (FG_AMM-01, 20)
	Quote 2: Acceptance of psychological disorders	“B: But don’t you think that the whole problem of why relatively little is offered or implemented in this area in the companies still has to do with stigmatisation? That people are afraid to address the psyche, especially supervisors and company owners? C: Yes, some of them certainly. But I think the hurdles are significantly lower than they were 5–10 years ago.” (FG_AMO-01, 280 et seq.)
“Advantages of PCIW”	Quote 3: Opportunities for the company and its employees	“And of course that’s great for us, because the employee returns to his job very quickly and is able to perform at full capacity again, and it is also satisfying for the employee, because he isn’t absent for very long, because he is available to his family again, and because he perceives the positive reaction very quickly.” (EM-03, 82)
	Quote 4: Opportunities for OPs	“(…) and on the other hand, we OPs are also becoming increasingly sensitised to such psychosomatic issues and are becoming more confident in recognizing them through this subsequent teaching or case review, quality circles, whatever you want to call it.” (FG_AMM-01, 34)
“Limitations of PCIW”	Quote 5: Intra-company limitations—Referral	“I don’t know if that was right, but I personally push people past because I don’t think the measure is effective and I don’t really believe that it works. Because if people don’t even understand ergonomic or manual handling guidelines, then how can a psychosomatic measure promise success? So that means that when these measures are offered, the threshold is very low, people are going to go, but sometimes my threshold needs to be adjusted or changed adequately.” (FG_AMM-01, 47)
	Quote 6: External limitations—Further treatment	**“**Yes, in principle it’s an interdisciplinary topic, a sector topic. Each agent in the healthcare sector is in his own world, the rehabilitation clinics, the outpatient therapists and we ourselves now have won over or been able to win over some, several external cooperation partners, increasingly more, who have now learned to work with this occupational setting. But the secondary institutions, that’s where it often breaks down (…).” (FG_AMM-01, 70)
	Quote 7: External limitations—not enough partners	Psychotherapists have much too little time to deal with this immense demand, and this relates to the psychotherapists’ training and also the focus, that this occupational focus even becomes a part of the training. Because it is really interesting that when you talk to these psychotherapists now, they talk about how for years the workplace hasn’t been recognized as playing an important role as a place of manifestation (FG_AMM_01, 65)
“Assessments and criticism of PCIW ”	Quote 8: No transfer of health insurance benefits	“(…) this is often something where an outsider trained in this can actually address the core of the problem and one can then find a solution. And this is also very effective with regard to the cost-benefit ratio. Also for the company. I am of the opinion, now with these multiple appointments, that the company or the revenue the company achieves should not be used to replace the services that would be required via the health insurance funds.” (FG_AMM-01, 30)
	Quote 9: Positive review	“I would find it helpful…. I can’t really estimate how many cases we have per year on the basis of a quantity structure. I would have to estimate that for you, but I would absolutely welcome the existence of such a range of services, in order to be able to fall back on a professional consultation, at least an initial consultation which would have to take place relatively quickly, either in acute cases or in cases where a mental disorder is anticipated.” (EO-01, 35-37)

Arabic numerals are used to mark the pseudonymization codes of the interview partners and the section in the MAXQDA file

EM, Individual interview with an expert with experience of psychosomatic consultations (PCIW); E0, individual interview with an expert without experience of PCIW; FG_AMM, focus group of OPs with experience of PCIW; FG_AM0, focus group of OPs without experience of PCIW

### Category: “Perspectives on common mental disorders (CMD) in the workplace”

In the opinion of the respondents interviewed and based on their experience, CMDs are becoming increasingly relevant disorders at the workplace. In terms of their origins, these disorders are perceived as multicausal—the result of a combination of personal and professional factors. The respondents consider the question of causality less important than that of finding a solution (Quote 1), since CMDs (also) manifest in the occupational context regardless of their cause and lead to a decrease in performance as well as periods of sick leave.

From their point of view, social acceptance of CMD is lower than that of somatic disorders, although there has been a noticeable increase in knowledge, awareness and acceptance regarding mental disorders in recent years (Quote 2).

### Category: “Advantages of the psychosomatic consultation in the workplace”

From the perspective of the respondents, PCIW allows employees quick access to a neutral, external specialist or psychotherapist, who can (at least initially) help them to deal with subjectively stressful and/or conflictual personal issues. For companies, the psychosomatic consultation allows employees with CMD to be identified as early as possible and may offer early treatment, meaning that long periods of absenteeism and the related costs can be avoided (Quote 3). Cases in which issues within the company are suspected to impact the development of CMD may especially profit from the model of PCIW: the close contact between psychotherapeutic counsellor and OP enables the psychotherapist to gain access to specific insights into the company. In these cases, the OP may significantly contribute to bridging the gap between the company and the consulting physician. Finally, this exchange can sensitize OPs to mental disorders, thereby allowing for (even) faster detection of CMD (Quote 4), intermediately and in the long run.

### Category: “Limitations of the psychosomatic consultation in the workplace”

PCIW reaches its intrinsic limits if there is a failure to implement necessary structural changes in the workplace, in cases where mental stressors are clearly associated with working conditions. A further limitation discussed is that some employees with a need for PCIW do not or cannot take advantage of it despite the low-threshold nature of the services offered. The latter may be the case for two reasons, both of which concern those authorized to make referrals (e.g. other health associated professionals within companies): either they feel competent to fill the role of the psychotherapeutic counsellor themselves or they assume that an employee would not benefit from PCIW and therefore do not refer him for the consultation (Quote 5).

The existing deficit in—especially outpatient—psychotherapeutic/psychosomatic standard care in Germany is discussed as an extra-occupational limitation of PCIW. This makes it difficult for employees in need of further treatment to find a continuing psychotherapeutic treatment (Quote 6). In addition, there are an insufficient number of psychotherapists and psychosomatic specialists available who focus on the professional work environment. This can complicate the search for collaborative partners who have sufficient capacity for taking on new patients (Quote 7).

### Category: “Assessments and criticism of the psychosomatic consultation in the workplace”

Fundamental, conceptual criticism has also been expressed with regard to PCIW. For this category, those aspects which are related to the perceived opportunities and limitations of the service offered will be presented below. Additional aspects of the programme are outlined in Preiser et al. (2014).

In the interviews, the point is made that the availability of PCIW can put pressure on employees to take advantage of such services when offered by the company. In addition, respondents also voice the criticism that PCIW could serve an “alibi function”. This occurs, for example, when there is a failure to implement structural changes on the basis of feedback of PCIW users. PCIW may also seem to make a promise for sufficient psychotherapy that is not honoured due to a lack of follow-up after the consultation. A few psychotherapeutic sessions may not be sufficient to profoundly help the employee and may serve more to improve the image of the company rather than to really support the employee. By contrast, another criticism raised states that the PCIW as an employee service should not aim to replace health insurance benefits (Quote 8).

Overall, however, the interviews contain rather little criticism and a high level of satisfaction among experts with experience with PCIW and reflect great interest on the part of experts with no prior experience in cooperating with such services (Quote 9).

## Discussion

### Key findings

Qualitative data obtained from two samples of occupational health physicians and stakeholders in companies in Baden-Württemberg, a highly industrialized state, revealed their notion that CMDs are becoming increasingly relevant disorders within the workplace setting, and that social acceptance of CMD is still lower than that of somatic disorders. Based on this background, their feedback suggests that PCIW allows employees quick access to a neutral, external specialist or psychotherapist and thus can help to avoid chronification of mental disorders. For companies, this may bear the advantage that longer periods of absenteeism (as well as presenteeism) can be avoided. They also feel that the ongoing collaboration with a psychotherapeutic specialist may sensitize OPs to identify mental disorders earlier and provide basic treatment. However, one limitation of PCIW is that it cannot replace necessary structural changes in the workplace, in case mental strain is primarily associated with working conditions. PCIW—if financed by the company—should also have clear time limits and cannot aim to replace health insurance benefits. Legislation and statutory health insurance companies should try to support regular outpatient psychotherapy after the initial consultation within PCIW, if such therapy is necessary. A further limitation discussed in the interviews is that some employees avoid the PCIW despite the low-threshold nature of the services offered. Overall, PCIW is perceived as an early, easy and fast first access to psychotherapeutic or psychosomatic specialist care. Thus, it may serve as a tool to further support social acceptance of mental disorders in society which at present is still perceived as on a low level (Henderson et al. 2014). As many individuals with CMD experience considerable stigma-related stress (Szeto and Dobson 2010), especially within the workplace setting, the offer of PCIW may serve as a valuable tool at the worksite to reduce stigma-related barriers due to mental illness, even though it is not an explicitly labelled anti-stigma intervention (Rüsch et al. 2014).

PCIW was developed and implemented by companies based on an anticipated need to support their employees and to reduce or, ideally, prevent the costs associated with CMD. Companies supply this opportunity against the background of a deficit in standard statutory care. Our data revealed a noteworthy limitation, however, as the PCIW can only reach its full potential in those for whom an intervention as funded by the specific company is sufficient to improve their health. This is in line with findings of one model offering brief psychotherapy (max. 8 sessions) directly linked to the PCIW, which revealed that for 40 % of patients, 6 sessions were satisfactory and they required no further therapy (Hölzer 2012). The second positive effect of PCIW, i.e. that patients requiring longer outpatient psychotherapeutic care can be motivated by PCIW to transition to standard statutory care, may be counteracted by the waiting times they face. This is consistent with actual data of waiting time in this part of the medical system in Germany (Kruse and Herzog 2012). However, PCIW or other company offers could serve to bridge the time until access to ordinary care is possible.

What interest do companies have in PCIW and to what extent are they responsible for such services? The majority of companies take many precautions to ensure their employees’ health and well-being at work. In addition, they follow a financial logic and are interested in keeping absenteeism and reduced performance among their workforces to a minimum. As described in the section “*limitations of the psychosomatic consultation in the workplace”*, our findings stress that the basic approach is to establish working conditions that allow (also) mental stress to be minimized as a primary preventive measure. Another approach is behavioural prevention, for example through health promotion measures, which, in terms of mental stress, include courses in topics such as stress management. In order to save further costs arising from reduced performance and absenteeism as described internationally (OECD 2014), some companies now also finance access to psychotherapeutic or psychosomatic specialists through PCIW (Mayer et al. 2010; Hölzer 2012; Preiser et al. 2014; Rothermund et al. 2014). There is already some evidence that any improvement in symptoms of CMD counts with regard to productivity (Jain et al. 2013) or work ability (Wåhlin et al. 2013) taking into account that work ability may stand for functional, especially social recovery (Pachoud et al. 2010, Buist-Bouwman et al. 2004). These companies take the initiative because they do not count on quick solutions by the standard statutory health system. The majority of individual interview, focus group and workshop participants considered corresponding services offered by companies as comprehensible in this respect. At the same time, they discussed the question of the responsibility of companies—some feared that such consultations fulfilled an alibi function and some generally rejected the idea of companies offering psychotherapeutic consultation and treatment as the statutory health system bears responsibility for providing those interventions.

Ultimately, companies face the challenge of structuring PCIW in a way that offering services of this kind supports their employees with CMD in their ability to work and perform without infringing on the area of responsibility of the health insurance bodies. German politics are now also rising to the challenge: the German Federal Ministry of Labour and Social Affairs, together with the social partners, aims at ensuring timely care for employees with CMD as part of the German Federal Government’s demographic strategy. One goal is to “improve the collaboration of social security institutions, both among themselves and with employers, in order to provide early care and support to workers with mental disorders and to reintegrate them quickly into working life” (BMAS 2013)—a concept shown to be efficient e.g. in Denmark or the Netherlands (Goorden et al. 2014). The PCIW model could be a starting point for this as it seems to provide early, low-threshold treatment.

### Strengths and limitations

Individual and focus group interviews with experts proved to be an appropriate data collection method for the present study to ensure rapid generation of extensive data on attitudes and preferences concerning PCIW. Saturation of the study population was not achieved, due to the time limitations of the project. Yet, various perspectives were included in the sample in order to reach data saturation even with a small study population. It is possible that new aspects might have been included in the data if additional experts had been interviewed. For example, it is imaginable that other experts would have put less emphasis on the OP as a key figure in the process. Since recurring themes emerged during the analysis, it can reasonably be surmised that the data represent central themes.

The evaluations of PCIW overall reflect a high level of user satisfaction throughout the interviews; there was very little fundamental criticism. This could also indicate that the study may have contained a bias. Perhaps willingness to participate was higher among OPs who had some experience of PCIW if this experience was positive. It is also possible that the interviewees who had some experience with the services had played a considerable part in initiating or structuring the services offered and therefore voiced fewer criticisms and doubts. Since the OPs without PCIW experience were found through (teaching) collaborations of the Institute of Occupational Medicine in Tübingen, it is conceivable that we reached particularly dedicated OPs who are very open-minded with regard to the topic. However, we were unable to obtain reliable information on this based on our data.

### Implications for practice

In our view, there are two prospective points that should be noted and embedded conceptually: firstly, the interface with the workplace and working conditions and secondly, the interface with or transition into outpatient mental health care.

It became clear from the interviews with those operating within the workplace that—in the worst case—an exclusively person-centred PCIW could serve an alibi function within the company, namely when it would not also be used for aggregate feedback and suggestions concerning work-related mental hazards within the respective company. In positive cases, the OP, the employer or both could serve as the point of contact for the consultant to give relevant feedback on dysfunctional working conditions—while still respecting the requirements of anonymity and medical confidentiality. In this case, the instrument of PCIW would not only serve to support individual workers but would also aim at various levels of prevention—individual and organizational (working conditions).

The second point of interface is that of external care. In addition to services within the company, such as family, debt and social counselling, external care consists mainly of early outpatient psychotherapeutic treatment services. In the future, it is conceivable that the psychosomatic medical consultant offering the PCIW or the (appropriately qualified) OP will be involved in the targeted return to work process, as already suggested by international models of collaborative care (Martin et al. 2012; Goorden et al. 2014; Arends et al. 2014).

## Conclusion

Close cooperation between those operating within the workplace—especially company-based OPs and other professionals—and external physicians and psychologists can overcome the hitherto rather strict division between care offered in the working environment and standard care. This is perceived as worthwhile by OPs who are involved in exchanges with medical consultants for the purposes of PCIW. From a European perspective, Henderson suggested that “our current approach of taking an ill person away from work, trying to make them better, then guessing at when they may be ready to return seems inflexible and unhelpful in comparison” (Henderson et al. 2011). PCIW seems to be a promising alternative approach trying to diagnose and treat employees with (beginning) CMD as early possible and while they are still actively working.

The innovative concept of anchoring psychotherapeutic consultations within an occupational setting is important and still under-researched. Thus, ongoing research investigates experiences and attitudes of the individuals affected by CMD and experiencing the new offer in a first qualitative examination, as well as its impact on the selection of the target group and the possible effectiveness of the concept (Rothermund et al. 2012).
